# Changes in the NK Cell Repertoire Related to Initiation of TB Treatment and Onset of Immune Reconstitution Inflammatory Syndrome in TB/HIV Co-infected Patients in Rio de Janeiro, Brazil—ANRS 12274

**DOI:** 10.3389/fimmu.2019.01800

**Published:** 2019-08-13

**Authors:** Carmem Beatriz Wagner Giacoia-Gripp, Andressa da Silva Cazote, Tatiana Pereira da Silva, Flávia Marinho Sant'Anna, Carolina Arana Stanis Schmaltz, Tania de Souza Brum, Juliana Arruda de Matos, Júlio Silva, Aline Benjamin, José Henrique Pilotto, Valeria Cavalcanti Rolla, Mariza Gonçalves Morgado, Daniel Scott-Algara

**Affiliations:** ^1^Laboratory of AIDS and Molecular Immunology, Oswaldo Cruz Institute (FIOCRUZ), Rio de Janeiro, Brazil; ^2^Clinical Research Laboratory on Mycobacteria, National Institute of Infectious Diseases Evandro Chagas (FIOCRUZ), Rio de Janeiro, Brazil; ^3^HIV Clinical Research Center, Nova Iguaçu General Hospital (HGNI), Rio de Janeiro, Brazil; ^4^Clinical Research Laboratory on Health Surveillance and Immunization, National Institute of Infectious Diseases Evandro Chagas (FIOCRUZ), Rio de Janeiro, Brazil; ^5^Platform for Clinical Research, National Institute of Infectious Diseases Evandro Chagas (FIOCRUZ), Rio de Janeiro, Brazil; ^6^Unit of Lymphocyte Cell Biology, Pasteur Institute, Paris, France

**Keywords:** TB/HIV Co-infection, NK cell repertoire, IRIS, innate immune cells, clinical outcomes

## Abstract

Tuberculosis (TB) is the most common comorbidity and the leading cause of death among HIV-infected individuals. Although the combined antiretroviral therapy (cART) during TB treatment improves the survival of TB/HIV patients, the occurrence of immune reconstitution inflammatory syndrome (IRIS) in some patients poses clinical and scientific challenges. This work aimed to evaluate blood innate lymphocytes during therapeutic intervention for both diseases and their implications for the onset of IRIS. Natural killer (NK) cells, invariant NKT cells (iNKT), γδ T cell subsets, and *in vitro* NK functional activity were characterized by multiparametric flow cytometry in the following groups: 33 TB/HIV patients (four with paradoxical IRIS), 27 TB and 25 HIV mono-infected subjects (prior to initiation of TB treatment and/or cART and during clinical follow-up to 24 weeks), and 25 healthy controls (HC). Concerning the NK cell repertoire, several activation and inhibitory receptors were skewed in the TB/HIV patients compared to those in the other groups, especially the HCs. Significantly higher expression of CD158a (*p* = 0.025), NKp80 (*p* = 0.033), and NKG2C (*p* = 0.0076) receptors was detected in the TB/HIV IRIS patients than in the non-IRIS patients. Although more NK degranulation was observed in the TB/HIV patients than in the other groups, the therapeutic intervention did not alter the frequency during follow-up (weeks 2–24). A higher frequency of the γδ T cell population was observed in the TB/HIV patients with inversion of the Vδ2^+^/Vδ2^−^ ratio, especially for those presenting pulmonary TB, suggesting an expansion of particular γδ T subsets during TB/HIV co-infection. In conclusion, HIV infection impacts the frequency of circulating NK cells and γδ T cell subsets in TB/HIV patients. Important modifications of the NK cell repertoire were observed after anti-TB treatment (week 2) but not during the cART/TB follow-up (weeks 6–24). An increase of CD161^+^ NK cells was related to an unfavorable outcome. Despite the low number of cases, a more preserved NK cell profile was detected in IRIS patients previous to treatment, suggesting a role for these cells in IRIS onset. Longitudinal evaluation of the NK repertoire showed the impact of TB treatment and implicated these cells in TB pathogenesis in TB/HIV co-infected patients.

## Introduction

Tuberculosis (TB) is one of the world's deadliest diseases and affects ~10.0 million people worldwide. A total of 1.3 million deaths occurred in 2017, and almost one-fourth of the global population is infected with the infectious agent of TB, *Mycobacterium tuberculosis* (*Mtb*) (1). TB is a leading killer of HIV-infected people (TB/HIV co-infection) worldwide (2). Brazil is a World Health Organization high-burden country for TB and TB/HIV-associated comorbidities (1). In 2017, almost 70.0 thousand TB cases were registered in the country, of which 6,501 (~9.4%) were TB/HIV cases. A total of 9.4% of these cases occurred in the city of Rio de Janeiro, which is ranked as the second Brazilian state capital in terms of the number of TB/HIV cases and is considered a TB-endemic region (3–5). Indeed, estimates from 2016 showed a higher TB incidence rate in Rio de Janeiro city than the national incidence (99 vs. 33.5 TB cases/100,000 hab) (3–5). TB/HIV co-infection poses enormous clinical, scientific, and public health challenges. Although the use of combined antiretroviral therapy (cART) during TB treatment improves patient survival, particularly by restoring immune functions, simultaneous management of cART and anti-TB drugs is not easy due to pharmacological interactions. Moreover, the appearance of an inflammatory syndrome called immune reconstitution inflammatory syndrome (IRIS) in some patients and early mortality are adverse events of cART initiation and represent challenges for the management of TB/HIV patient care (6, 7). IRIS has been associated with lower CD4^+^ T cell counts at cART initiation followed by a dramatic improvement in these cell counts and a rapid plasma viral load decay that lead to partial restoration of overall immune functions, and the presence of opportunistic pathogens (8–11); however, the immunopathogenesis of this syndrome and the major clinical and laboratory factors associated with IRIS onset are not clearly understood (12). In TB/HIV IRIS patients, elevated concentrations of proinflammatory serum mediators have been detected at IRIS onset, including C-reactive protein and cytokines, such as IL-6, IL-12, and TNF (13–15). Following cART initiation, recovery of CD4 Th1 responses to mycobacterial antigens is reported, and these increased responses have been associated with IRIS (16–18). Indeed, in a previous study by our group, an increase in immune responses was observed in TB/HIV IRIS patients compared with those of non-IRIS patients, as was a marked increase in IFN-γ production by T cells from IRIS patients in response to PPD and other *Mtb* antigens (19).

Given their importance in antigen processing and pathogen trafficking, cells of the innate immune system are a focus of increasing interest in IRIS physiopathology. γδ T cells appear to play a predominant role against *Mtb* infection, and one study demonstrated reduced numbers of inhibitory natural killer (NK) receptors on mycobacteria-specific Vδ2^+^ γδ T cells in TB/HIV IRIS patients (17). Moreover, studies have examined NK cell function in the development of IRIS among TB/HIV patients. In a study of unmasking IRIS, these cells were found to express increased levels of activation markers (20). In a longitudinal study with a TB/HIV co-infected group in Cambodia, NK cells isolated from paradoxical IRIS patients had higher expression of the degranulation marker CD107a than those of non-IRIS patients prior to IRIS onset at a time point 2 weeks after initiating TB treatment but before starting cART (21). The authors hypothesized that increased NK cell-mediated lysis of *Mtb*-infected cells might increase the antigen load and the risk of IRIS occurrence. Therefore, the physiopathology of TB/HIV coinfection and the occurrence of IRIS might be explained by an exaggerated innate immune response, implicating the participation of different players as Toll-like receptors, inflammasomes, NK cells, monocytes/macrophages, neutrophils, cytokines, among others (21–26).

Moreover, among TB patients, HIV infection is a risk factor for extrapulmonary TB, which sometimes require a high index of suspicion for diagnosis. Although TB is acquired by inhalation of contaminated droplets, it can produce disease in any organ system in addition to the lungs, which are usually the initial site of infection. In the context of TB/HIV co-infection, an increase in the number of reported cases of extrapulmonary TB is observed, accounting for ~20% of TB cases in HIV-uninfected patients and 53–62% of TB cases among HIV-infected patients (27). In our previous studies, ~56% of extrapulmonary TB cases were registered in our cohort of TB/HIV patients, including disseminated cases (19, 28). Although the association between HIV and overall TB (including pulmonary TB) is well-characterized (29), the relationship between extrapulmonary TB and HIV is less clear; the mechanisms related to the escape of *Mtb* to TB sites out of the lungs are not yet fully clarified, although extrapulmonary TB is very likely due to the reduction of CD4^+^ T cell counts in HIV-infected patients, since CD4^+^ T-helper cells are important for controlling of *Mtb* infection (30–34).

In this scenario, we hypothesized that the association between the exaggerated responses of NK cells before TB treatment and cART and the increased risk of IRIS after starting both therapies, as observed in the TB/HIV patients from Cambodia (21), would also be found in populations with different genetic backgrounds, such as patients from Rio de Janeiro city, Brazil. We also hypothesized a differential implication of innate immunological parameters in the pathogenesis of extrapulmonary TB, affecting lymph nodes and disseminated TB cases. Therefore, this study aimed to explore the implications of innate immune responses, specifically NK cell responses, in the evolution of TB/HIV co-infection.

## Methods and Patients

### Study Population

The primary study population consisted of 33 HIV-infected patients diagnosed with TB (TB/HIV) who started cART 2 weeks (W2) after TB treatment according to the Brazilian Guidelines for HIV/AIDS Treatment and the National Tuberculosis Program (Ministry of Health) at the time of the study (35, 36). Briefly, 4-drugs TB regimen containing rifampicin, isoniazid, pyrazinamide, and ethambutol, followed by the first line HIV treatment with two nucleoside reverse transcriptase inhibitors (NRTI) + non-nucleoside reverse transcriptase inhibitors (NNRTI) (35–37). Tuberculosis diagnosis was made when suggestive clinical symptoms, and radiological findings were present. In some cases, microbiological methods confirmed the diagnosis: Ziehl-Neelsen staining for the detection of acid-fast bacilli in sputum smears and biopsy specimens, Xpert MTB/RIF® in sputum for MTB and rifampicin resistance detection, mycobacteria culture of sputum, and biopsy samples in Löwenstein-Jensen medium and/or Mycobacteria Growth Indicator Tube (MGIT) (36). HIV infection was determined by rapid test and confirmed by conventional serology testing and western blot, and then, molecular detection of RNA viral load (35). As inclusion criteria, the TB/HIV patients were naïve for HIV drugs and presented CD4^+^ T cell counts below 200 cells/mm^3^. The TB/HIV patients were evaluated at the introduction of anti-TB therapy at the baseline visit (D0), at the initiation of cART (W2) and during the clinical follow-up visits at weeks 6, 10, 14, and 24. Four TB/HIV patients developed paradoxical IRIS defined as a documented clinical worsening of TB signs or symptoms during anti-TB treatment, after cART initiation, not explained by any other diseases or by an adverse effect of drug therapy; lymph node enlargement with inflammatory signs temporally related to cART introduction was considered IRIS in this study (37–39). Each case of IRIS was validated in this study by the members of the clinical coordination team to discard opportunistic disease diagnosis, drug-resistant TB, low adherence, or adverse effects of cART. The TB/HIV patients were also analyzed according to their TB clinical presentations as pulmonary (PTB), lymph node TB (LNTB), and disseminated TB (DTB) (when two non-contiguous sites were affected) (37). Moreover, in the context of clinical outcomes, the patient outcomes were evaluated as favorable (TB treatment responders) or unfavorable (death) at the end (usually 6 months) of TB therapy under cART. In parallel to the TB/HIV patients, three control groups were also studied: HIV mono-infected subjects (HIV; *n* = 25), who were evaluated previous to cART; HIV-seronegative TB patients (TB; *n* = 27), who were evaluated previous to TB treatment; and volunteer healthy controls (HC, *n* = 25). The diagnosis of HIV infection was discarded by conventional serology and HIV RNA viral quantification in TB and HC groups, while TB infection was ruled out by the clinic and radiological examination in HIV and HC groups. However, if healthy participants had any sign or symptom, Xpert MTB/RIF® in sputum for MTB and rifampicin resistance detection and mycobacteria culture of sputum samples in Löwenstein-Jensen medium and/or MGIT were performed. All participants, including the HC, who agreed to participate in this study were enrolled after signing the informed consent form. Participant recruitment started in August 2014 and extended until December 2016 at the Instituto Nacional de Infectologia Evandro Chagas (INI), which is the clinical research unit for adult care in infectious diseases of the Oswaldo Cruz Foundation, and at the Hospital Geral de Nova Iguacu (HGNI), which is a general hospital from the Brazilian Ministry of Health. HGNI is located in the outskirts of Rio de Janeiro, and is a referral center for HIV and TB care in 13 municipalities. This protocol was approved by the local institutional Ethics Committee (Instituto Oswaldo Cruz Research Ethics Committee) and the Brazilian National Ethics Committee—CONEP (CAAE: 04514012.1.1001.5248).

### Phenotypic Innate Lymphoid Cell Studies

NK cells, invariant NKT cells (iNKT) and γδ T cell subsets were characterized from fresh whole blood through multiparametric flow cytometry (Gallios flow cytometer and Kaluza Version 3.0 analysis software, Beckman-Coulter, Kendon, FL, USA). NK cells were identified as CD3^−^CD56^+/−^CD16^+/−^ within the gated lymphocyte population previously defined in the FSC/SSC dot plot. The same strategy was used to define the iNKT cells, which were identified using the monoclonal antibody 6B11, specific to the conserved CDR3 region of the canonical hallmark T cell receptor (TCR)-invariant chain Vα24Jα18 (40–42) and defined as CD3^+^CD56^+/−^Vα24Jα18^+^ lymphocytes, as detailed in the Supplementary Figure 1A. In its turn, the γδ T cell subsets were identified as CD3^+^CD56^+^Panγδ^+^ lymphocytes coexpressing or not the Vδ2 chain. The NK repertoire receptors were evaluated using monoclonal antibodies based on the CAPRI NK study results (21), including the KIR family (CD158a, CD158b1/b2,j, CD158d, CD158e, CD158e1/e2, and CD158i), NCR receptors (NKp30, NKp44, and NKp46), NKG family (2A, 2C, and 2D), CD94, CD85j, CD160, CD161, NKp80, DNAM-1, CD244, and activation marker CD69 (from Beckman-Coulter; BD Biosciences, San Jose, CA, USA; R&D Systems, Minneapolis, MN, USA; and BioLegend, San Diego, CA, USA).

### Cytokine Assays and Cytolytic NK Cell Activity

CD107a expression on NK cells and the intracellular cytokine staining (ICS)-based assay were used to measure cytotoxic activity (degranulation) and cytokine expression as described previously with some modifications (21, 43). Briefly, mononuclear cells (PBMCs) isolated from fresh whole blood were incubated overnight at 37°C with 5% CO_2_ at 1:1 ratio with the K562 cell line, which was used as target cells, in the presence of a CD107a monoclonal antibody, brefeldin A (5 μg/mL; Sigma-Aldrich, St. Louis, MO, USA) and monensin (6 μg/mL; Sigma-Aldrich), which were added concomitantly to the cells. Spontaneous basal degranulation and cytokine production were measured by incubating PBMCs under the same conditions without target cells. After incubation, anti-CD3, anti-CD56, and anti-CD16 were added, followed by antibodies for the ICS-based assay (IFN-γ, TNF, TGF-β, and IL-10; Beckman-Coulter and BD Biosciences). These evaluations were assessed by multiparametric flow cytometry using the XL-MCL and Gallios equipment from Beckman-Coulter.

### Statistical Analysis

The HIV viral load and immunological parameters, including CD4^+^ T cells, NK cells (receptor repertoire, CD107a expression, and cytokine levels), iNKT and the γδ T innate cell subsets, were assessed in the TB/HIV patients during follow-up and in the control group at baseline (D0). Descriptive analyses of clinical and demographic characteristics were performed for the patients and controls. The Wilcoxon *t*-test, Mann-Whitney *U* test, and Kruskal-Wallis test were used to evaluate quantitative variables, whereas Fisher's exact test and Pearson's chi-square test were used for categorical variables. A *p*-value < 0.05 was considered significant. The slope of the CD4^+^ T cell absolute count and HIV viral load was estimated by a non-linear least squares method for a five-parameter sigmoidal curve (44). Briefly, the slope indicates the growth/degrowth rate of an indicator during the “window of linearity,” which is the highest linearity region of the curve. The maximum and minimum points of the second derivate curve were used as boundaries for this region. The statistical analysis was performed using the Stata software version 11.0 (STATA Corp. College Station, TX, USA), and the GraphPad Prism version 6.0 software (GraphPad Software, La Jolla, CA, USA) was used to generate the graphics. Sigmoidal fitting was performed using R version 3.0.3 (2014-03-06—“Warm Puppy”).

## Results

### Demographic Characteristics of the Studied Groups

The clinical and sociodemographic characteristics obtained from all participants in the study are presented in Table 1. No differences in age, gender, race or tuberculosis presentation were observed in the distribution of participants among the groups, although a trend for a higher frequency of TB occurrence was observed for males. The basal CD4^+^ T cell absolute counts were extremely low among the TB/HIV and HIV patients compared to those of the other participants (*p* = 0.0001). As expected, the basal CD4/CD8 ratios were also lower among these patients than in the TB and HC groups (*p* = 0.0001). Interestingly, the TB/HIV group presented HIV viral loads that were significantly higher than those of the HIV group (*p* = 0.0077). The major TB presentation was pulmonary regardless of the presence of HIV coinfection, followed by lymph node TB. Disseminated TB was diagnosed in 15.1% of the TB/HIV patients, whereas no case was detected in the TB group. Among the 33 TB/HIV recruited participants, only 4 (12.1%) developed symptoms consistent with IRIS after beginning cART, which was in agreement with the frequency of IRIS observed in our previous studies (28, 45). The IRIS onset was observed from 1–3 weeks after cART initiation and was clinically diagnosed as defined above. In general, the patients presented worsening of the signs/symptoms related to TB, like fever and enlargement of the right or the left cervical/submandibular lymph nodes, which were either self-resolving, or the patients were treated with corticoid-based therapy, such as Prednisone (Supplementary Table 1). At D0, the IRIS and non-IRIS TB/HIV patients presented similar CD4^+^ T cell absolute counts (data not shown). Concerning the HIV viral load, higher levels were observed for the IRIS patients at the D0 than for the non-IRIS TB/HIV participants (*p* = 0.0088). As defined previously, 26 (78.8%) of the 33 TB/HIV patients were classified as having a favorable outcome, 5 (15.1%) were defined as having an unfavorable outcome, and outcome information were missing for 2 (6.1%) at the end of the study follow-up.

**Table 1:** Clinical and sociodemographic characteristics of the TB/HIV, HIV, and TB patients and HC volunteers.

**Characteristic**	**TB/HIV** **(*N* = 33)**	**HIV** **(*N* = 25)**	**TB** **(*N* = 27)**	**HC** **(*N* = 25)**	*P*-value^*^
Age (years), median (IQR)	35 (32–42)	36 (27–43)	36 (27–48)	44 (34–49)	0.1433^a^
**Gender**, ***n*** **(%)**					
Male	24 (72.7)	17 (68.0)	17 (63.0)	10 (40.0)	0.063^b^
Female	9 (27.3)	8 (32.0)	10 (37.0)	15 (60.0)	–
**Race**, ***n*** **(%)**					
Black	6 (18.2)	4 (16.0)	4 (14.8)	3 (12.0)	0.102^c^
Brown	16 (48.5)	15 (60.0)	11 (40.7)	9 (36.0)	–
White	11 (33.3)	6 (24.0)	12 (44.4)	13 (52.0)	–
**Site of tuberculosis**, ***n*** **(%)**					
Pulmonary	20 (60.6)	na	20 (74.1)	na	0.213^c^
Lymph node	6 (18.2)	na	5 (18.1)	na	–
Extrapulmonary	2 (6.1)	na	2 (7.4)	na	–
Disseminated	5 (15.1)	na	–	na	–
**IRIS**, ***n*** **(%)**					
Yes	4 (12.1)	na	na	na	–
No	29 (87.9)	na	na	na	–
**Clinical outcomes**, ***n*** **(%)^e^**					
Favorable	26 (78.8)	na	27 (100)	na	–
Unfavorable	5 (15.5)	na	–	na	–
Missed	2 (6.1)	na	–	na	
Baseline CD4 (cells/mm^3^), median (IQR)	57 (17–144)	42 (23–139)	745 (537–980)	1118 (872–1,320)	0.0001^a^
Baseline CD4/CD8 ratio median (IQR)	0.08 (0.04–0.17)	0.11 (0.05–0.19)	1.41 (1.00–2.07)	1.88 (1.57–2.38)	0.0001^a^
Baseline HIV-1 viral load (log_10_ copies/ml), median (IQR)	5.64 (5.22–5.88)	5.28 (4.99–5.59)	na	na	0.0077^d^

*N, number of cases; IQR, Interquartile ranges (75th−25th percentiles); IRIS, Immune Reconstitution Inflammatory Syndrome; na, not applicable*.

a *Kruskall-Wallis test*.

b *Chi-square test*.

c *Fisher's exact test*.

d *Mann-Whitney U-test*.

e *Clinical outcomes analysis is only related to TB*.

* *p < 0.05*.

### Virological and Immune Responses to TB and/or cART Treatment During Follow-Up

**Figure 1:**
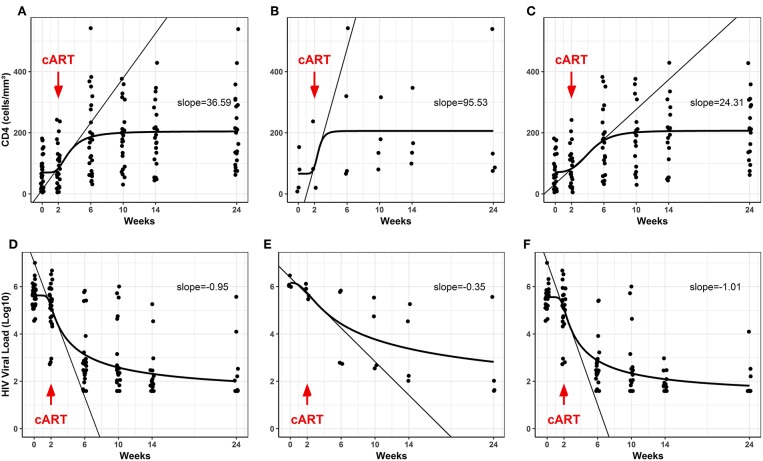
Blood CD4^+^ T counts and plasma HIV viral loads in the TB/ HIV patients during follow-up. **(A–C)** Evolution of CD4^+^ T cell counts (absolute values) and **(D–F)** the HIV viral load (Log_10_ copies/mL) after starting cART (red arrows) in the TB/HIV **(A,D)**, IRIS **(B,E)**, and non-IRIS **(C,F)** patients. The results are presented as the slopes of the CD4^+^ T cell (cells/mm^3^) increase and HIV viral load (Log_10_ copies/mL) decrease.

The CD4^+^ T cells and HIV viral load were also evaluated for the TB/HIV group at starting TB treatment (D0), at cART initiation 2 weeks later (W2), and during the follow-up visits (W6, 10, 14, and 24) (Figure 1). The TB/HIV patients showed a significant increment of CD4^+^ T cells after the first month of cART (at week 6), and this increase was sustained over the follow-up (Figure 1A; *Slope* = 36.59). Notably, a higher slope was detected for the CD4^+^ T cell increment for the TB/HIV IRIS patients (Figure 1B; *Slope* = 95.53) than for the non-IRIS patients (Figure 1C; *Slope* = 24.31), which indicated the higher increase in CD4^+^ T cell counts among the IRIS patients. As expected, the HIV viral load significantly declined from weeks two to six, for which a negative slope of 0.95 was observed, and this decay was continuous during the follow-up (Figure 1D). A similar response was observed when the TB/HIV non-IRIS patients were assessed separately, implying the development of fast control of HIV replication with cART in these patients, whereas less prominent control of the viral load was detected in the IRIS patients (Figure 1E: IRIS *Slope* = −0.35, Figure 1F: non-IRIS *Slope* = −1.01).

### Circulating Innate Immune Cells and the NK Cell Repertoire Profile Before Clinical Intervention

**Figure 2:**
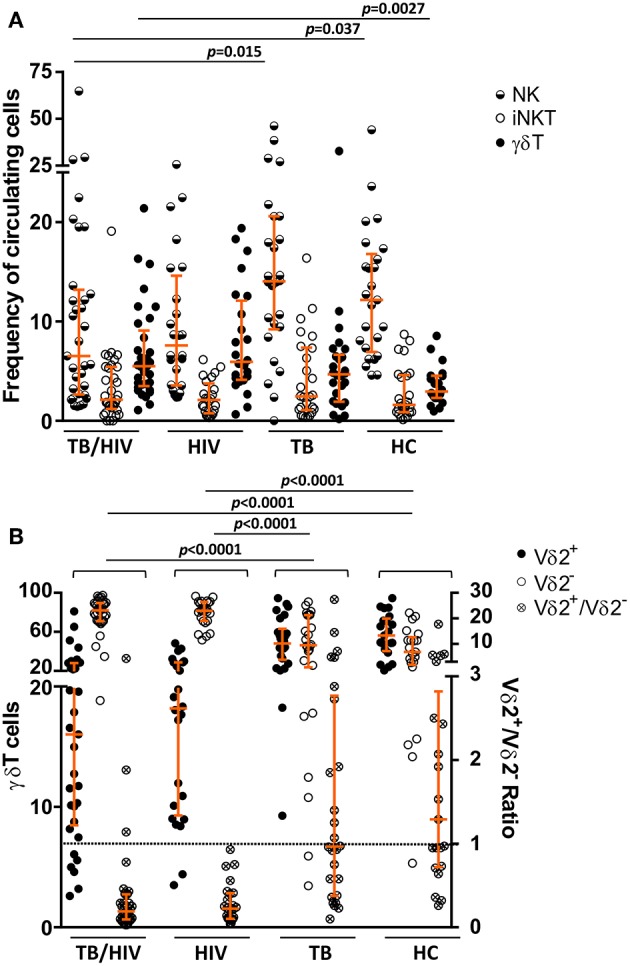
NK, iNKT, and γδ T cell subset phenotyping. The NK, iNKT, and the γδ T cell subsets were analyzed prior to TB treatment and/or cART. The frequencies of **(A)** the circulating NK, iNKT (CD3^+^CD56^+^ Vα24Jα18^+^) and γδ T cell populations, **(B)** the Vδ2^+^ and Vδ2^−^ T cell subsets, and the Vδ2^+^/Vδ2^−^ ratio for TB patients co-infected with HIV (TB/HIV, *n* = 33), HIV mono-infected individuals (HIV, *n* = 25), TB-only patients (TB, *n* = 27), and healthy controls (HC, *n* = 25) at D0. The results are expressed as the % of total lymphocytes **(A)** or γδ T cells **(B)**. Histograms represent the median and interquartiles (25th−75th percentile) for each group. A dashed line indicates the Vδ2^+^/Vδ2^−^ ratio = 1.0. The data were analyzed using the Mann-Whitney *U* test. Significant *p*-values (*p* < 0.05) are indicated.

To characterize the circulating NK, iNKT and γδ T cell profiles, fresh whole blood samples collected from all participants were investigated at the D0. The TB/HIV patients presented a lower frequency of CD56^+/−^CD16^+/−^CD3^−^ NK cells than the healthy controls (*p* = 0.037) and TB patients (*p* = 0.015), showing the negative impact of HIV infection on circulating NK cells (Figure 2A). Concerning the iNKT cells, no difference in the frequencies of CD3^+^CD56^+^Vα24Jα18^+^ cells was identified among the TB/HIV patients and the other groups (Figure 2A). The same scenario was observed when the CD3^+^Vα24Jα18^+^ iNKT population was analyzed among the groups (Supplementary Figure 1B). Moreover, higher percentages of circulating γδ T cells were observed in the TB/HIV patients compared to those of the HC participants (*p* = 0.0027; Figure 2A). The frequencies of γδ T cells expressing the Vδ2 chain (Vδ2^+^) were decreased in the TB/HIV and HIV-infected patients compared to those in the HCs and TB patients. As expected, γδ T cells expressing other Vδ chains (Vδ2^−^) were the predominant subset among the TB/HIV and HIV mono-infected patients, with a very low Vδ2^+^/Vδ2^−^ ratio (Figure 2B; *p* < 0.0001).

**Figure 3:**
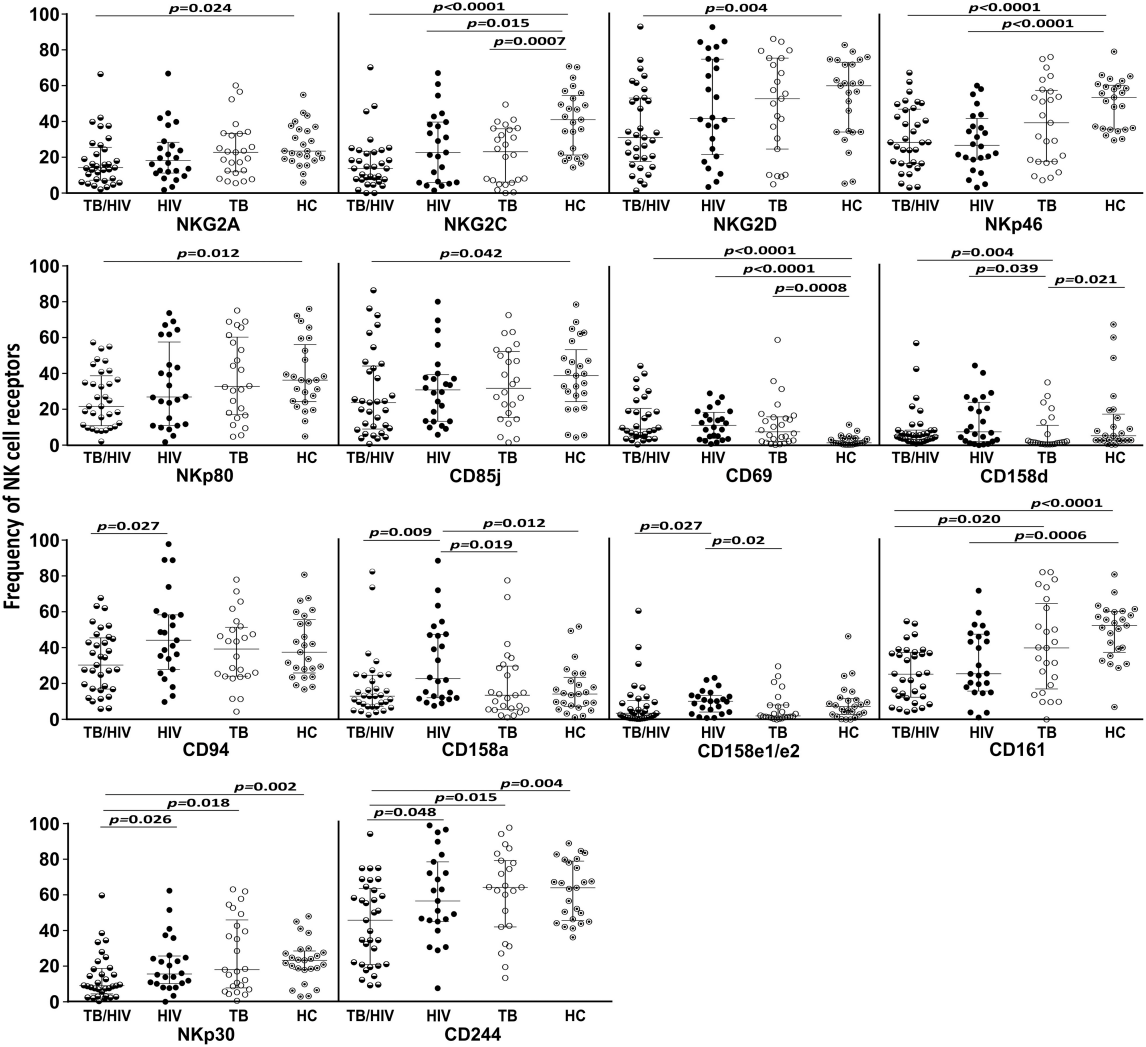
NK cell repertoire before TB and/or cART treatment according to the groups. The results are shown as frequencies of NK cell receptors among the total NK cell population of TB patients co-infected with HIV (TB/HIV, *n* = 33), HIV mono-infected individuals (HIV, *n* = 25), TB-only patients (TB, *n* = 27) and healthy controls (HC, *n* = 25) at D0. The study receptors were C-type lectin receptors (NKG2A, NKG2C, NKG2D, NKp80, CD94, CD69, and CD161), natural cytotoxicity receptors (NKp30 and NKp46), killer cell immunoglobulin-like receptors (CD158d, CD158a and CD158e1/e2), immunoglobulin-like transcript receptor CD85j, and the costimulatory CD244 molecule. The results are presented as the median and interquartiles (25th−75th percentile). The data were analyzed using the Mann-Whitney *U* test. Significant *p*-values (*p* < 0.05) are indicated.

Concerning the NK cell repertoire, we tested 20 different receptors. The results for those presented statistically significant differences among the TB/HIV patients and the other groups are depicted in Figure 3. Except for CD158d and CD69, generally, the TB/HIV patients showed lower frequencies of these selected NK receptors than the other studied groups. These alterations targeted important activating and inhibitory molecules indistinctly and showed the impact of both comorbidities on the NK cell repertoire. Medians and interquartile ranges for all investigated receptors for all groups are presented in Supplementary Table 2.

### Analysis of NK Subsets in IRIS Patients

**Figure 4:**
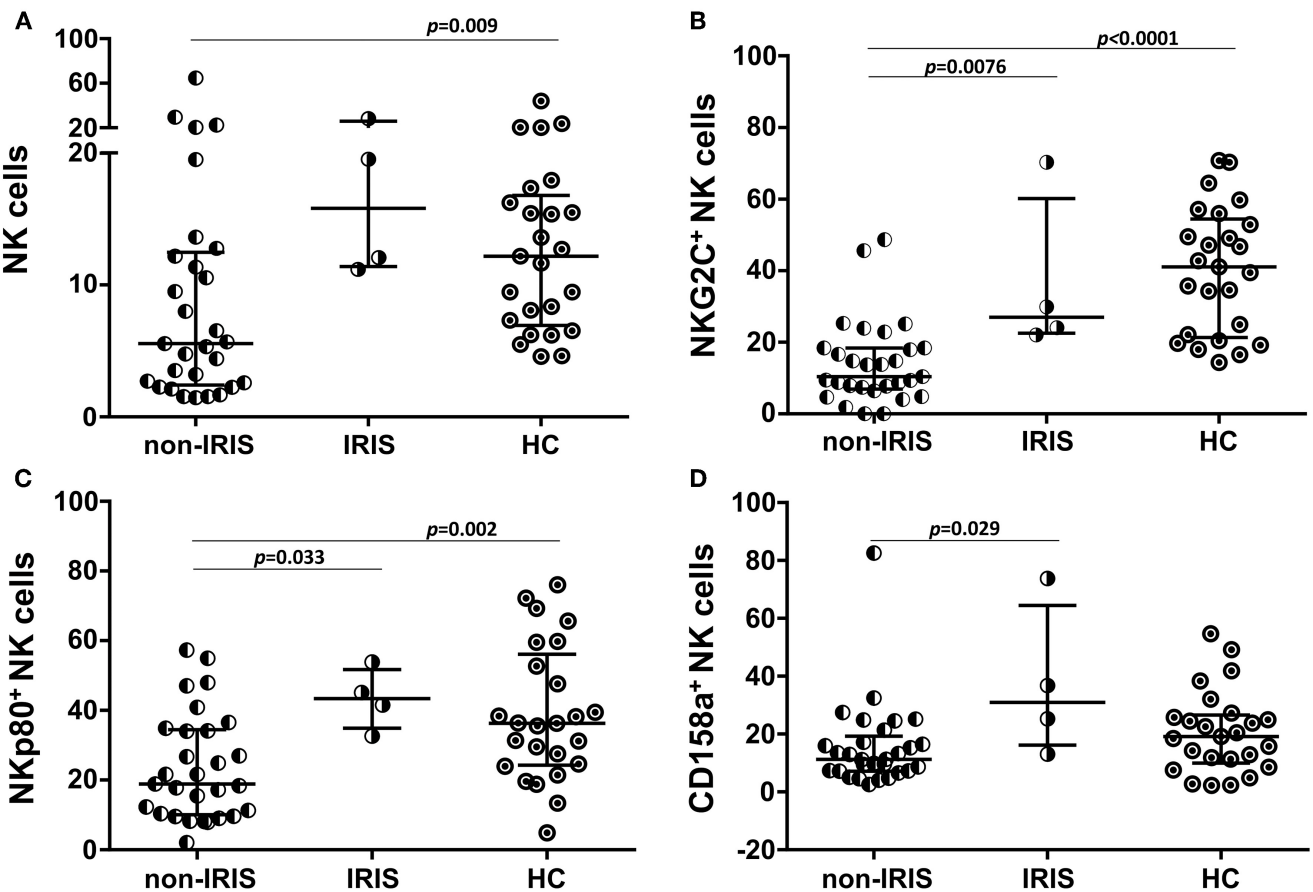
Expression of NK cell receptors in IRIS and non-IRIS patients prior to TB treatment and cART. Different frequencies observed between the TB/HIV IRIS (*n* = 4) and non-IRIS (*n* = 29) patients for **(A)** circulating NK cells and NK subsets expressing **(B)** NKG2C, **(C)** NKp80, and **(D)** CD158a compared to those of the healthy control (HC, *n* = 25) group. The results are presented as the median and interquartiles (25th−75th percentile). The data were analyzed using the Mann-Whitney *U* test. Significant *p*-values (*p* < 0.05) are indicated.

The impacts of the NK subsets on the inflammatory process were assessed in the TB/HIV IRIS patients despite the low number of confirmed IRIS cases. Prior to TB treatment, the non-IRIS patients presented a lower frequency of NK cells than the HC (*p* = 0.009), and a trend for a lower frequency compared to that of the IRIS patients (*p* = 0.055). Conversely, the IRIS patients and HCs had more similar NK cell frequencies (Figure 4A). Interestingly, three NK receptors (NKG2C, NKp80, and CD158a, Figures 4B–D) were decreased in the non-IRIS patients but were expressed at similar levels between the IRIS patients and HC. At IRIS onset, no significant difference was found in the NK cell subsets between the patients with and without IRIS (data not shown). Despite the limited number of IRIS cases, these observations suggest a more preserved NK cell profile among the IRIS patients, similar to the HC. The iNKT and γδ T cells were also evaluated, but no significant differences were observed for these populations between the IRIS and non-IRIS patients at study entry.

### Frequencies of Innate Immune Cells in TB/HIV Patients According to the TB Clinical Presentation

**Figure 5:**
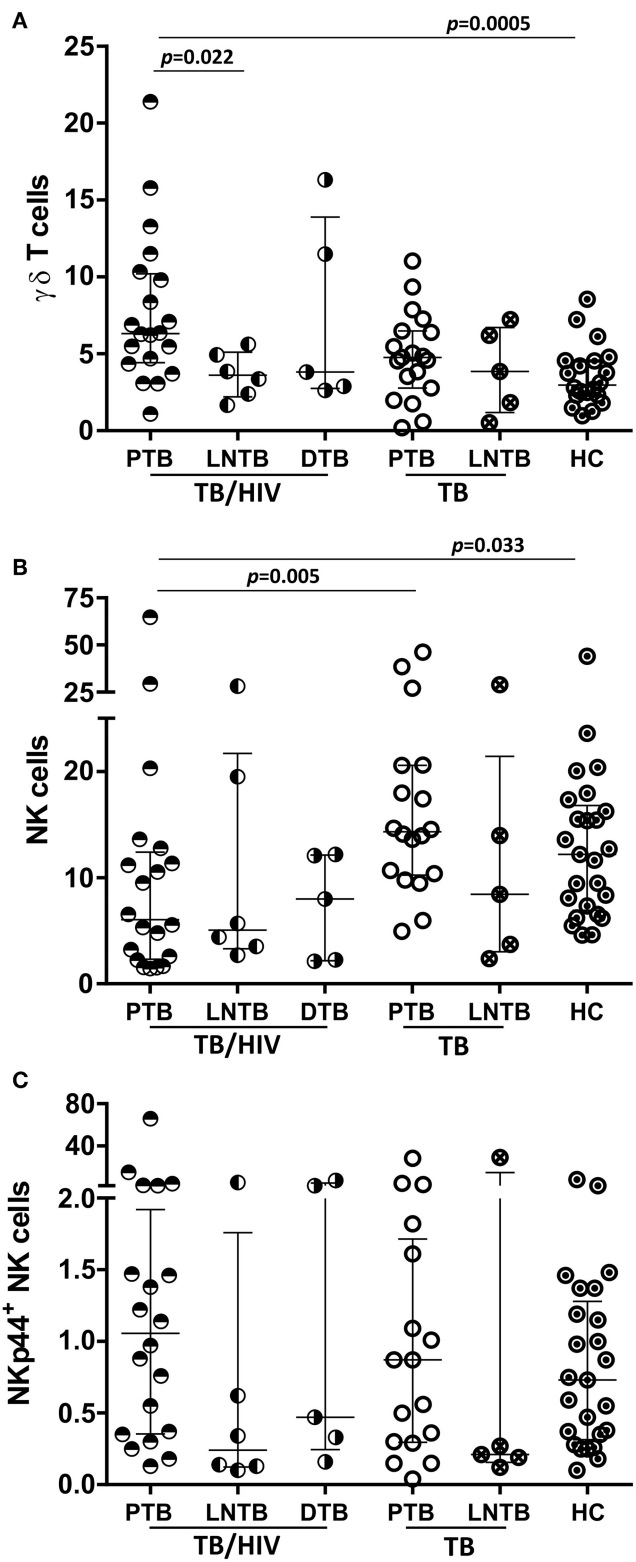
Frequencies of NK and γδ T cells according to the TB clinical presentation. Percentages of **(A)** circulating γδ T cells **(B)** NK cells and **(C)** the NKp44^+^ NK subset (among the total NK cells) in the TB/HIV and TB groups according to pulmonary (PTB: TB/HIV, *n* = 20; TB, *n* = 20), lymph node (LNTB: TB/HIV, *n* = 6; TB, *n* = 5) or disseminated (DTB: TB/HIV, *n* = 5) clinical presentations of tuberculosis and compared to those of the healthy controls (HC, *n* = 25). The results are presented as the median and interquartiles (25th−75th percentile). The data were analyzed using the Mann-Whitney *U* test. Significant *p*-values (*p* < 0.05) are indicated.

The innate lymphocyte subsets were also analyzed in the context of the TB clinical presentation regardless of the onset of IRIS. TB/HIV patients with PTB presented higher γδ T cell frequencies than either the HC (Figure 5A; *p* = 0.0005) or TB/HIV patients presenting LNTB (*p* = 0.022), whereas no difference was observed in those with DTB (Figure 5A). These differences were not detected between the TB patients presenting with PTB and LNTB. A trend for a higher γδ T cell frequency was observed in the TB/HIV patients compared to that of the TB patients (Figure 5A; *p* = 0.056) with PTB. These observations suggested that γδ T cell expansion occurred in cases of co-infected patients with PTB presentation. Concerning the NK cell frequency, a decrease was observed for PTB cases among the TB/HIV patients compared to that of the HC (*p* = 0.033) and the TB patients presenting with PTB (Figure 5B; *p* = 0.005). The NKp44^+^ NK cell frequency tended to be higher in the TB/HIV patients presenting with PTB than with LNTB, suggesting that more *in vivo* activation of NK cells occurred in this group of patients (Figure 5C; *p* = 0.056). The same findings were not observed between the TB PTB and LNTB groups, reinforcing the role of HIV infection in NK cell activation. No significant differences were observed in either iNKT cells or other profiles of NK subsets when they were analyzed according to the TB clinical presentation (data not shown).

### Potential for NK Cell Degranulation and Cytokine Production

**Figure 6:**
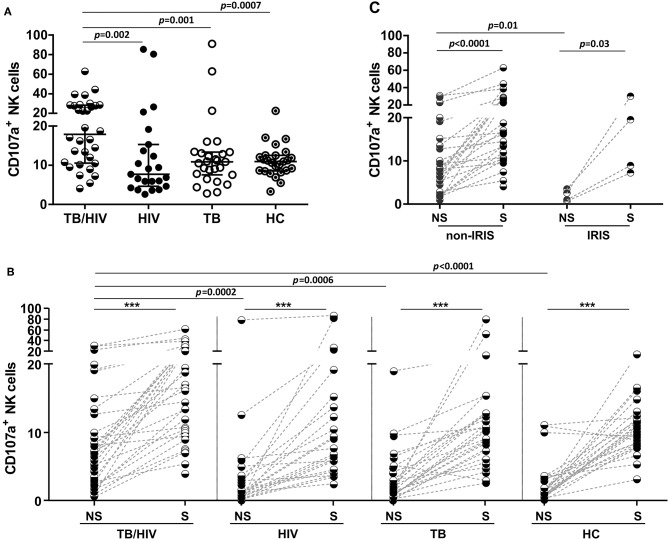
Degranulation capacity of NK cells before TB and/or cART treatment. Frequencies of CD107a^+^ NK cells at D0 in **(A)** TB/HIV co-infected patients, HIV mono-infected (HIV) patients, TB cases (TB) and healthy controls (HCs) after stimulation with the target cell line, **(B)** in TB/HIV IRIS and non-IRIS patients, and **(C)** under non-stimulated (NS) and stimulated (S) conditions with K562 target cells. The results are shown as **(A)** the median and interquartiles (25th−75th percentile). The data were analyzed using the Mann-Whitney *U* test, for intergroup analysis or the Wilcoxon *t*-test for intragroup analysis. Significant *p*-values (*p* < 0.05) are indicated. ***p < 0.0001.

The potential for NK cell degranulation and cytokine production was also investigated among the groups. NK cell degranulation was addressed through CD107a expression; the results are shown in Figure 5. A significantly higher percentage of NK cells expressing CD107a was observed in the TB/HIV patients at D0 than among the HIV, TB, and HC groups (Figure 6A). However, when we analyzed the degranulation capacity of NK cells according to the development of IRIS, no difference was observed between the IRIS patients and the other groups. Moreover, no difference was observed in CD107a-expressing NK cells in the TB/HIV patients regardless of the TB clinical presentation (data not shown). We also analyzed CD107a expression on NK cells under stimulated and non-stimulated conditions. *In vitro* stimulation with the target cell line was able to significantly increase the frequency of CD107a^+^ NK cells in all groups compared to that of the non-stimulated condition (Figure 6B; *p* < 0.0001). Furthermore, significantly higher CD107a^+^ NK cell percentages were detected in the TB/HIV patients under the non-stimulated condition than in the other groups, indicating *in vivo* NK cell activation. Interestingly, this higher CD107a expression without stimulation was not observed more frequently in IRIS patients (Figure 6C; *p* = 0.01). Both the IRIS and non-IRIS patients had similarly increased CD107a^+^ NK cell frequencies when stimulated *in vitro* by target cells (Figure 6C).

**Figure 7:**
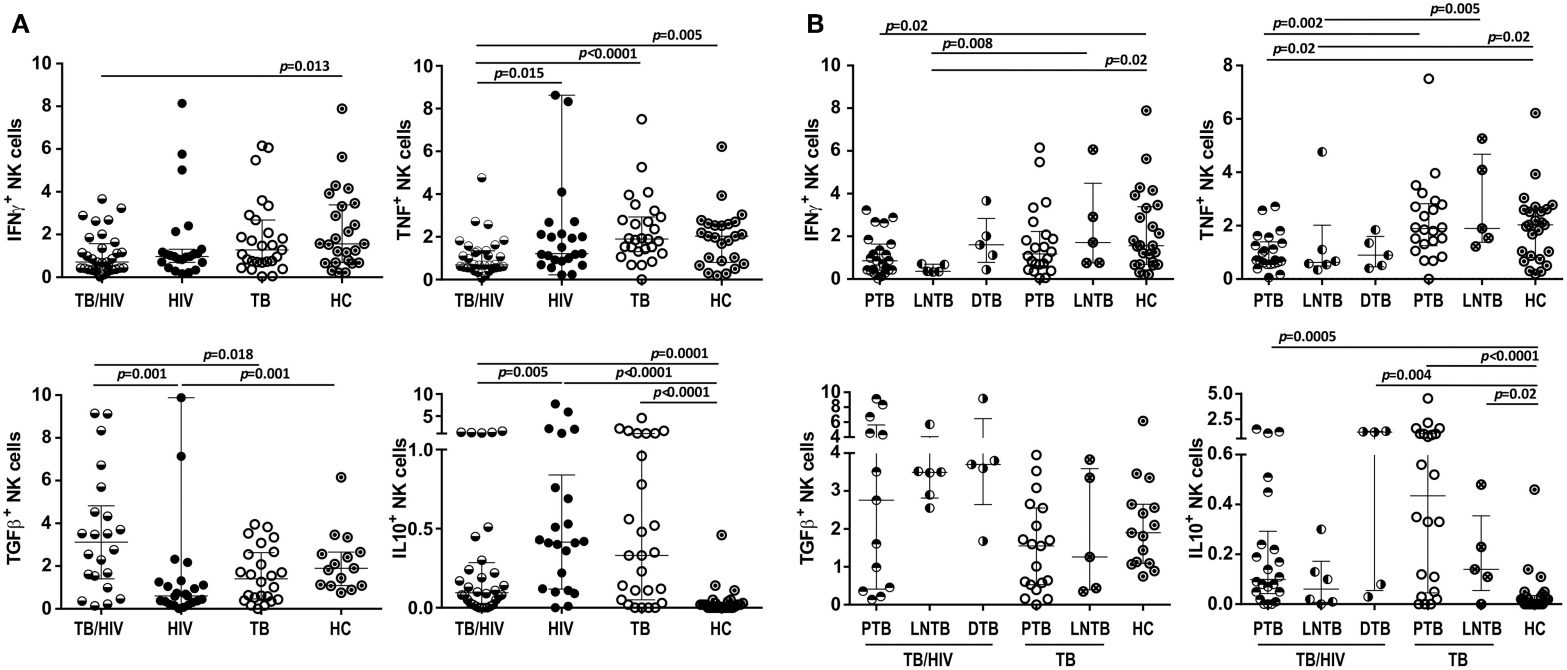
Frequencies of NK cells producing intracellular cytokines under *in vitro* stimulation. Percentages of IFN-γ^+^, TNF^+^, TGF-β^+^, and IL-10^+^ NK cells after stimulation with target cells at D0 in **(A)** the TB/HIV co-infected, HIV mono-infected (HIV), TB-only (TB) and healthy control (HC) groups, **(B)** according to the TB clinical presentation as pulmonary (PTB), lymph node (LNTB), and disseminated TB in TB/HIV co-infected patients and for PTB and LNTB in the TB-only patients. The results are presented as the median and interquartiles (25th−75th percentile). The data were analyzed using the Mann-Whitney *U* test. Significant *p*-values (*p* < 0.05) are indicated.

Intracellular IFN-γ, TNF, TGF-β, and IL-10 cytokine production by NK cells was assessed after *in vitro* stimulation with target cells. As shown in Figure 7A, the TB/HIV patients presented a decrease in IFN-γ and TNF staining compared to that of the HC and the HIV, TB, and HC groups, respectively. On the other hand, the frequency of TGF-β-producing NK cells in the TB/HIV group was significantly higher than that in the HIV mono-infected and TB patients. Despite the very low frequencies of IL-10^+^ NK cells observed in all groups, higher production was observed in TB- and/or HIV-infected patients compared to that of the HC. No differences were observed when intracellular cytokine production was compared between the IRIS and non-IRIS patients (not shown). By contrast, lower IFN-γ and TNF production were detected in the PTB and LNTB TB/HIV patients than in the HC (Figure 7B), whereas the PTB and DTB TB/HIV patients had higher levels of IL-10^+^ NK cells than the HC. Furthermore, IFN-γ- and TNF-producing NK cells were also significantly lower in the LNTB-TB/HIV patients than in the LNTB-TB and the PTB and LNTB TB/HIV patients, respectively. No specific TGF-β production profile was observed regardless of the TB clinical presentation.

### Impacts of TB Treatment on the NK Repertoire

**Figure 8:**
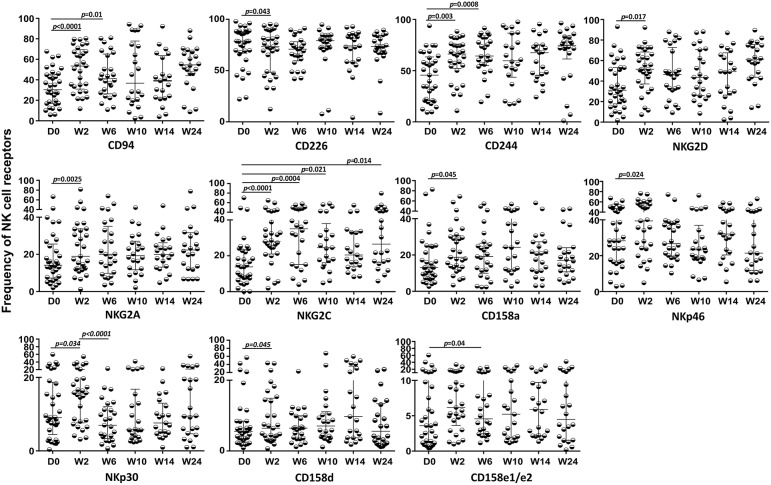
Expression of NK cell receptors during follow-up in the TB/HIV co-infected patients. Expression of different NK receptors in the TB/HIV patients before and after treatment intervention (anti-TB at day 0 and cART at week 2) and at weeks (W) 2, 6, 10, 14, and 24. The results are represented as the median and interquartiles (25th−75th percentile). The data were analyzed using the Wilcoxon *t*-test. Significant *p*-values (*p* < 0.05) are indicated.

Despite a satisfactory virological response and quantitative recovery of CD4^+^ T cells, no significant recovery of circulating NK, iNKT, and γδ T cells, including the Vδ2^+^ and Vδ2^−^ subsets, was detected during the follow-up (data not shown). However, significant alterations were observed in the NK cell repertoire after the first 2 weeks of TB treatment (W2) and prior to cART for almost all receptors, except for NKG2C, which also presented significant changes throughout the 24 weeks of follow-up, as presented in Figure 8. Some receptor frequencies were also altered with cART, including CD158e1/e2, which presented significant changes in its frequency only after cART initiation. No differences were observed during the follow-up in TB/HIV patients evaluated according to the TB clinical forms (data not shown).

### NK Cell Subsets Associated With an Unfavorable Clinical Outcome

**Figure 9:**
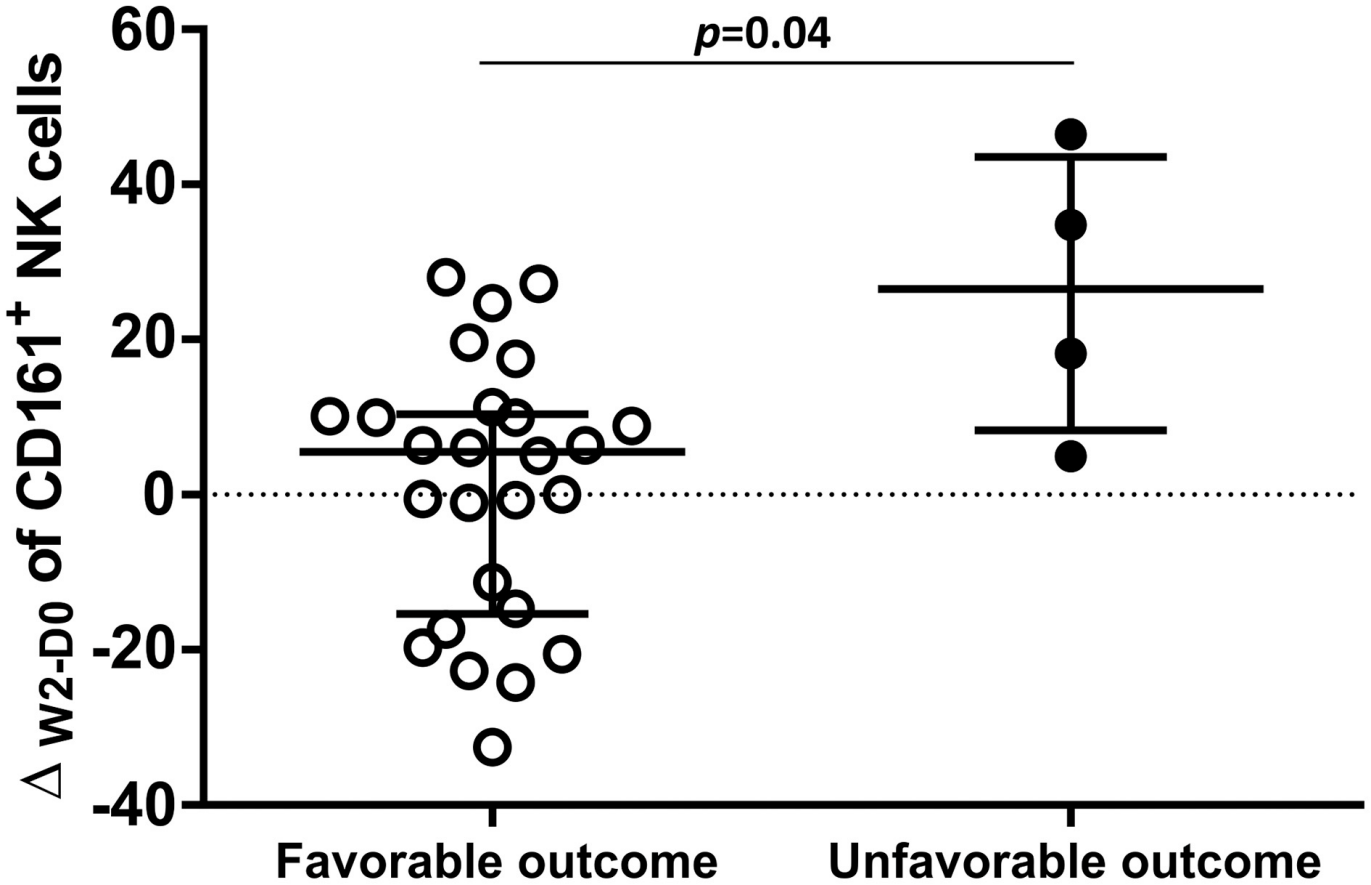
Distribution of Δ_W2−D0_ for CD161^+^ NK cells. Frequencies obtained for the differences in CD161^+^ NK cell expression between D0 and week 2 (W2) in TB/HIV patients analyzed based on favorable (*n* = 26) or unfavorable (*n* = 5) clinical outcomes. The results are presented as the median and interquartiles (25th−75th percentile). The data were analyzed using the Mann-Whitney *U* test. A significant *p*-value (*p* < 0.05) is indicated.

We hypothesized that some association would exist between the NK cell repertoire changes at W2 of TB treatment and the patients' clinical outcomes. The TB/HIV patient outcomes were classified as favorable in cases of TB treatment responders or unfavorable in cases of death, which were the two clinical outcomes observed in our study group. Then, we calculated the delta between D0 and W2 (Δ_W2−D0_) for all NK cell receptors according to the clinical outcomes. A significant difference was only observed for the circulating CD161^+^ NK cell subset, which presented a significantly higher Δ_W2−D0_ in the TB/HIV patients with an unfavorable clinical outcome (Figure 9).

### NK Cell Degranulation and Cytokine Production During Treatment Follow-Up

**Figure 10:**
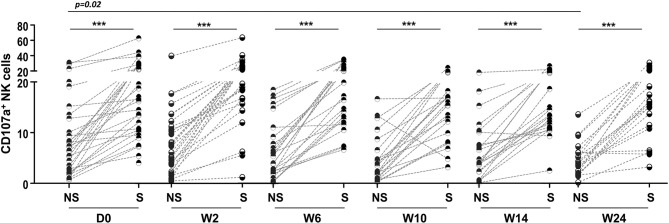
Degranulation capacity of NK cells during the study follow-up. Frequencies of CD107a^+^ NK cells detected in TB/HIV co-infected patients before and after treatment intervention under non-stimulated (NS) or stimulated (S) conditions with K562 target cells. The data were analyzed using the Wilcoxon *t*-test. Significant *p*-values (*p* < 0.05) are indicated. ***p < 0.0001.

Higher frequencies of CD107a^+^ NK cells were observed in TB/HIV patients after stimulation with target cells at all follow-up visits compared to those of the HIV, TB and HC groups at D0 despite TB treatment and cART (*p* < 0.05, data not shown). As observed at D0, *in vitro* stimulation of NK cells with target cells induced higher CD107a^+^ NK cell expression at all follow-up visits (Figure 10). CD107a expression in cultures without stimulation tended to be lower during the follow-up and became significantly different at W24 (Figure 10; *p* = 0.02) compared to that at D0. TB and/or cART treatment did not appear to impact significantly the NK granulation capacity after stimulation, at least during the first 24 weeks. Finally, longitudinal analysis of the ICS frequency among the TB/HIV patients revealed no changes during follow-up (data not shown).

## Discussion

In the present work, we investigated the innate blood lymphocyte profile, specifically NK cells, in TB/HIV patients residing in Rio de Janeiro city, Brazil, who were cART naive and evaluated the implications for IRIS development, TB clinical presentation, and clinical outcomes. The HIV/TB association poses enormous clinical challenges, starting with an early, and accurate diagnosis, especially for extrapulmonary cases. Additionally, this association includes the provision of effective anti-TB treatment, use of concurrent cART, management of drug cytotoxicity and IRIS, including its treatment in some cases (46).

In the absence of therapeutic intervention, the NK cell repertoire is impacted by co-infection, since TB/HIV patients present alterations in several NK cell receptors that mainly include decreases in expression. A skewed NK repertoire has been observed in HIV infection, and these alterations have even been associated with HIV-1 pathogenesis and disease progression (47, 48). We also observed an impact of HIV infection, since HIV mono-infected patients also presented alterations in the NK repertoire frequencies compared with those of the healthy controls. As reported previously (49–52), modifications in the NK cell repertoire were observed in HIV-seronegative TB patients. These results highlighted the complex interactions of NK cells during TB and/or HIV infection.

In our cohort of TB/HIV patients, only four of 33 (12.1%) patients developed paradoxical IRIS after cART initiation despite their severe immunodeficiency (median CD4^+^ T cell absolute count: 57 cells/mm^3^; IQR: 17–144), and high HIV viral loads (median: 5.64 Log_10_ copies/mL; IQR: 5.22–5.88) at study inclusion, besides the early initiation of cART after starting TB therapy, which are considered significant risk factors for IRIS development (53, 54). The IRIS frequency in this study is in accordance with that reported in our previous studies (28, 45), but differs from the frequencies observed in other populations. This finding reinforces the concept that the IRIS incidence varies according to the geographic region (11), and can be explained by the inclusion of patients with different genetic backgrounds.

Although many studies of HIV/TB patients who developed IRIS have led to the determination of the most prominent risk factors for development of this syndrome, such as a low CD4^+^ T count before cART initiation followed by a successful CD4^+^ T cell increase (55, 56), the underlying mechanisms responsible for IRIS have not been clearly defined. One of the most explored mechanisms is its association with the expansion of antigen-specific Th1 CD4^+^ T cells shortly after cART initiation (16), although subsequent studies questioned whether this association could be considered truly causal (9). However, elements of the innate immune system, such as monocytes/macrophages, dendritic cells (DCs), neutrophils, γδ T lymphocytes, NK cells, and related soluble factors have also been implicated in the onset of IRIS, and have been the focus of important investigations (17, 21, 22, 24, 26, 57).

One clinical trial in Cambodia reported a significantly higher NK cell degranulation capacity in IRIS patients before cART and after 2 weeks of TB treatment than in non-IRIS patients, followed by decreased expression of NK cell activating receptors (NKp30, NKp46, and NKG2D) at IRIS onset (21). Although we diagnosed a small number of IRIS cases, we also identified alterations in the NK cell profile between the IRIS and non-IRIS patients. A decrease in the frequency of NK cells expressing NKp80 and NKG2C was observed in the TB/HIV non-IRIS patients, whereas these receptors were expressed at normal levels in the IRIS patients compared to those of the healthy controls. Moreover, the non-IRIS patients also presented a reduction of NK cells expressing the KIR receptor CD158a. Recently, NKp80 expression was closely related to NK cell functional maturity in secondary lymphocyte tissues, suggesting an important regulatory role for this receptor during the maturation process at those sites that were potentially related to the acquisition of NK cell functionality (cytotoxicity and/or cytokine production) (58). Thus, a loss of this marker could be related to lower NK cell functions in non-IRIS patients. However, we did not find a correlation between the degranulation capacity of NK cells, and expression of the NKp80 marker in the non-IRIS patients (results not shown). NK cells expressing the NKG2C activating receptor have been described as an NK memory-like population against multiple viral infections, especially cytomegalovirus but also viruses such as simian immunodeficiency virus (59–62). Moreover, the NKG2C^+^ NK population has been suggested to have adaptive properties against *Mtb* and to be able to discriminate between latent and active tuberculosis (increased proportions in latent tuberculosis) (63). The decrease in NKG2C^+^ NK cells observed in the non-IRIS patients suggests lower NK cell recognition of HIV- and/or TB-infected cells. When considered together with the decrease in NKp46 expression, the NK cell receptors implicated in lysis of TB-infected target cells (64) could explain the lower inflammatory response in this group of patients. In turn, the CD158a/KIR2DL1 receptor acquires its inhibitory function during the developmental licensing process of NK cells, and the absence of this type of receptor during NK cell development has been associated with less functional competence to respond to the lack of HLA ligands on a target cell (65, 66). By contrast, the expression levels of the above-cited receptors in IRIS patients are similar to those observed in the HC group (higher than in the non-IRIS patients) and might suggest a more preserved NK cell repertoire in IRIS patients, who could mount an increased inflammatory response.

In the present study, we also identified alterations in γδ T cells in TB/HIV patients similar to those observed in untreated individuals with chronic HIV-1 infections (67). Higher γδ T cell frequencies were observed in TB/HIV patients than in the healthy controls, with the expansion of the γδ T cell subset expressing the Vδ chain rather than Vδ2 and a low Vδ2^+^/Vδ2^−^ ratio. Although not observed in the present study, a higher frequency of Vδ2^+^ T cells was shown in TB/HIV IRIS patients compared to that of non-IRIS patients (17). Interestingly, when we analyzed γδ T cells and their relationship with the clinical presentations of TB, we identified greater mobilization of γδ T cells in TB/HIV patients presenting pulmonary TB that was not observed with either TB/HIV lymph node TB presentation or in the HCs and was almost higher than that in the HIV-seronegative TB patients. γδ T cells, especially Vδ2^+^ T cells, have been widely reported to play an important role in protective immune responses to *Mtb* infection. These cells are expanded early during *Mtb* infection and are inclusive in recently acquired latent infection (68–70). In non-human primates, these cells even attenuate TB pathology and contain lesions primarily in the infection site of the lung, with no or reduced TB dissemination (71). However, in our study, the γδ T population in the TB/HIV patients was predominantly composed of Vδ2^−^ cells, as previously reported. In our study, these cells may be engaged in the immune response against pulmonary TB presentation and may be important innate immune players involved in containment of *Mtb* infection in the lungs in the absence of therapeutic intervention.

In relation to another important lymphocyte population involved in regulatory/effector functions, the iNKT cells (40–42, 72), no significant differences were observed in the present study when TB/HIV patients were compared with the control groups (TB, HIV and HC), as well among the TB clinical forms, onset of IRIS, TB outcomes, or throughout the 6 month of follow-up under TB-treatment and cART, differing from previous studies showing reduced frequency of iNKT cells in HIV, TB or TB/HIV patients compared with healthy controls (73, 74), and between IRIS and non-IRIS cases (74). Historically, the iNKT cells were originally identified with the Vα24 and Vβ11 mAb (75, 76), but it was later shown that the combination of these two selective reactivities did not formally define this population accurately (71, 77). In the present study, iNKT cells were accurately analyzed as Vα24Jα18^+^ cells, using the monoclonal antibody 6B11, in the context of the CD3^+^CD56^+^ population, as supported by vast literature (40–42, 78). However, it has been further shown that not all iNKT cells are CD56^+^ (72). In this sense, we re-analyzed the Vα24Jα18^+^ cells in the context of the CD3^+^ population and, similarly, the obtained results did not show significant differences in the frequency of this population among the studied groups and/or clinical conditions.

We also investigated the *in vitro* degranulation and cytokine production potential of NK cells from TB/HIV patients. We observed a higher degranulation capacity in TB/HIV patients than in the other groups. Different from the results obtained in the study in Cambodia (21), we did not observe differences in the CD107a^+^ NK cell frequencies between the IRIS and non-IRIS patients after *in vitro* stimulation with a target cell line or not. However, the results from Cambodia were obtained after 2 weeks of TB treatment and before cART initiation, whereas in our study, the degranulation capacity was assessed before any treatment intervention. Moreover, we did not detect an increase in degranulation capacity of NK cells after 2 weeks of TB treatment or later (during the follow-up under cART and TB treatment). Although *in vivo* NK cell activation was observed in our study groups (based on an increase in some activation markers and degranulation without any activation) similar to that in the Capri NK study (21), we should consider other factors, such as differences either on genetic backgrounds and/or local endemic pathogens able to stimulate innate immunity. Interestingly, spontaneous CD107a^+^ expression was significantly lower in the IRIS than in the non-IRIS patients. The spontaneous NK cell degranulation observed in the non-IRIS TB/HIV patients may result from a skewed NK repertoire. Consistent with this observation, the lower frequency of CD158a^+^ NK cells observed in these patients could be related to a more immature profile among these individuals compared to the more regulated and lower CD107a^+^ NK cell expression levels detected in the IRIS patients.

Although NK cells regulate immune responses through cytokines secretion (79), there are few reports describing the NK regulatory cytokine secretion patterns in TB/HIV patients (80). In our study, TB/HIV patients showed a lower frequency of intracellular IFN-γ-producing NK cells compared to the HC group, as well as of TNF- compared to HIV, TB, and HC groups. On the other hand, the frequency TGF-β- or IL-10-producing NK cells in the TB/HIV group was significantly higher than that in the HIV and TB patients. These results reflect the interplay between pro-inflammatory and regulatory cytokines in TB/HIV patients and could indicate the establishment of regulatory mechanisms to control the exacerbated inflammatory response by the innate immune system in TB/HIV patients. Similar to our results, lower frequency of NK intracellular pro-inflammatory cytokines, IFN-γ and TNF-, were observed in TB/HIV patients from India compared to healthy controls (80).

We observed that several NK cell receptors were enhanced during the two first weeks of TB treatment. There is no report of NK cell repertoire modifications after 2 weeks of TB treatment in patients infected with TB/HIV or TB alone. Only one communication has studied modifications of NK receptors after 6 months of TB treatment in pulmonary TB (51). Although recovery of NK cell function was observed, perturbations of the NK cell repertoire were still present at the endpoint of the study (51). We do not have a full explanation for this discrepancy, but genetic factors may be differently implicated in these cohorts. For the TB/HIV study group, cART was started in the second week of TB treatment, and the patients were followed in the study until the end of TB treatment at week 24. Despite the CD4^+^ T cell recovery and control of HIV-1 replication, cART had a low or no impact on the NK cell repertoire. Similarly, other reports were not able to show the recovery of NK cells despite the long cART follow-up period (up to 85 months), with viral replication suppression and increased levels on CD4^+^ T counts (9). Moreover, recovery of the NK cell repertoire during cART has been described (81), but the restoration takes more time than the recovery of CD4^+^ T cells, in particular when the CD4^+^ T- counts are very low, like in our study. Our limited follow-up did not allow us the observation of late modifications in the NK cell repertoire and functions. However, early modifications induced during TB treatment alone showed that patients with an unfavorable outcome presented a higher frequency of CD161^+^ NK cells than patients with a favorable TB recovery outcome. CD161 is a marker of human IL-17-producing T cell subsets, and its expression marks pro-inflammatory NK cells with high cytokine responsiveness that may contribute to inflammatory disease pathogenesis (82–84). Induction of modifications such as those reported here was observed after IL-10 treatment of PBMCs, including induction of higher CD161 marker expression on NK cells (84, 85). Although the treatment schedule used in this study was related to a reduced risk of death (52), five of the 33 HIV/TB patients presented this unfavorable outcome, and the NK cells with a pro-inflammatory profile observed in these TB/HIV patients could have participated in this process.

Our study has some limitations. The main limitation was the low numbers of IRIS TB/HIV patients, patients with lymph node and disseminated TB presentation, and unfavorable outcomes/death, which possibly were due to the low number of TB/HIV patients recruited during the study period even though the city of Rio de Janeiro is an endemic region for tuberculosis. Indeed, the recruitment of a larger number of the TB/HIV patients, as initially planned, was not achieved along the period of the study support, possibly due to the implementation by the Brazilian Ministry of Health of the free early access to antiretrovirals in the recent years, independently of the CD4^+^ T-cell counts and clinical conditions, and also INH prophylaxis for some HIV-infected patients, restricting our access to participants with CD4^+^ T-cell counts <200 cells/mm^3^, a inclusion criteria to the TB/HIV and the HIV patients (86). Because of the sample size, multivariate analyses were not carried out, which restricted the statistical analysis to univariate analyses. Future investigations are necessary to prove the concepts observed and discussed here, such as the association of IRIS development in TB/HIV patients with a more conserved and specialized NK cell profile, the relationship of γδ T cells with the immune response in patients with pulmonary TB presentation, and the involvement of pro-inflammatory NK cells with an unfavorable outcome of TB/HIV coinfection.

## Data Availability

All datasets generated for this study are included in the manuscript and/or the Supplementary Files.

## Ethics Statement

This study was carried out in accordance with the recommendations of the Resolution 466/12 from the Health National Council from the Brazilian Ministry of Health with written informed consent from all subjects. The protocol was approved by the Instituto Oswaldo Cruz Research Ethics Committee and the Brazilian National Ethical Committee—CONEP.

## Author Contributions

CG-G, JP, MM, and DS-A conceptualized and designed the study and also contributed to the experimental design and provided intellectual input. CG-G, AC, and TdS performed sample processing and clinical data analysis and provided intellectual input. CG-G and AC performed the experiments. VR, JP, TB, FS, and CS enrolled the patients and provided clinical data. CG-G, JdM, and JS performed the statistical analysis. AB provided ethical and regulatory support. CG-G, MM and DS-A analyzed the data and wrote the manuscript. All authors reviewed and approved the final version of the manuscript.

### Conflict of Interest Statement

The authors declare that the research was conducted in the absence of any commercial or financial relationships that could be construed as a potential conflict of interest.

## Supplementary Material

Supplementary Figure 1 Definition of iNKT (Vα24Jα18^+^) cells on CD3^+^ T lymphocytes and on the CD56^+^CD3^+^ cells. **(A)** An initial gate defined the lymphocytes, in a forward scatter (FSC) vs. side scatter (SSC) dot plot. Then, a CD3^+^ gate was established in a CD56^+/−^ vs. CD3^+^ dot plot, and used to define the CD3^+^ iNKT cells in the Vα24Jα18^+^ *vs*. CD3^+^ dot plot. In parallel, the same CD3^+^ gate was used to gate the CD3^+^CD56^+^ cells, from which the CD3^+^CD56^+^ vs. Vα24Jα18^+^ dot plot defined the iNKT cells. **(B)** Comparative analysis of iNKT cells among the TB/HIV (*n* = 33), HIV (*n* = 25), TB (*n* = 27), and HC (*n* = 25) groups, before any therapeutic intervention, based on the CD3^+^CD56^+^Vα24Jα18^+^ or CD3^+^ Vα24Jα18^+^ populations.

Supplementary Table 1 Clinical characteristics of the TB/HIV IRIS patients.

Supplementary Table 2 Medians and interquartile ranges (IQRs) of the circulating NK cell repertoire frequencies.
