# Supplementary figures and images for: Changes in the NK Cell Repertoire Related to Initiation of TB Treatment and Onset of Immune Reconstitution Inflammatory Syndrome in TB/HIV Co-infected Patients in Rio de Janeiro, Brazil—ANRS 12274
Source: Front Immunol. 2019 Aug 13;10:1800. doi: 10.3389/fimmu.2019.01800 (PMC6700218; doi:10.3389/fimmu.2019.01800)

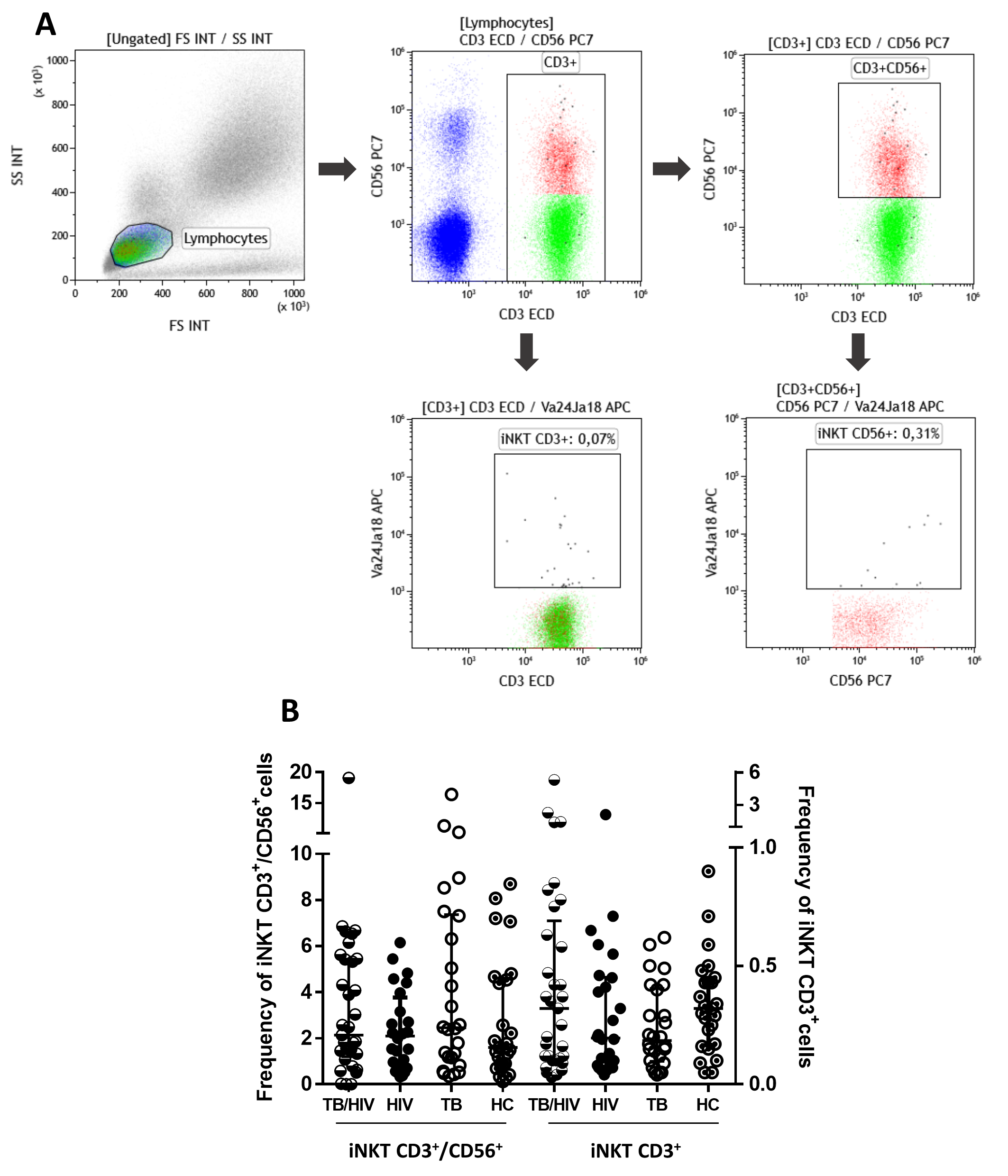

Supplement: Supplementary Figure 1 — Definition of iNKT (Vα24Jα18+) cells on CD3+ T lymphocytes and on the CD56+CD3+ cells. (A) An initial gate defined the lymphocytes, in a forward scatter (FSC) vs. side scatter (SSC) dot plot. Then, a CD3+ gate was established in a CD56+/− vs. CD3+ dot plot, and used to define the CD3+ iNKT cells in the Vα24Jα18+ vs. CD3+ dot plot. In parallel, the same CD3+ gate was used to gate the CD3+CD56+ cells, from which the CD3+CD56+ vs. Vα24Jα18+ dot plot defined the iNKT cells. (B) Comparative analysis of iNKT cells among the TB/HIV (n = 33), HIV (n = 25), TB (n = 27), and HC (n = 25) groups, before any therapeutic intervention, based on the CD3+CD56+Vα24Jα18+ or CD3+ Vα24Jα18+ populations. [file Image_1.TIF]
