# Supplementary material for: Changes in the NK Cell Repertoire Related to Initiation of TB Treatment and Onset of Immune Reconstitution Inflammatory Syndrome in TB/HIV Co-infected Patients in Rio de Janeiro, Brazil—ANRS 12274
Source: Front Immunol. 2019 Aug 13;10:1800. doi: 10.3389/fimmu.2019.01800 (PMC6700218; doi:10.3389/fimmu.2019.01800)
Supplement: Supplementary Table 1 — Clinical characteristics of the TB/HIV IRIS patients. [file Table_1.DOCX]

**Supplementary Table 1. Clinical characteristics of the TB/HIV IRIS patients**

|  |  |  |  |  |  |  |
| --- | --- | --- | --- | --- | --- | --- |
| **Patients** | **TB laboratorial diagnoses** | **Clinical sign and symptoms** | **TB clinical presentation** | **IRIS onset after starting cART (Days)** | **IRIS-related sign and symptoms** | **IRIS-management** |
| 002 | AFB sputum smear +, Xpert MTB/RIF^®^ + | Yes | Pulmonary | 17 | Left cervical lymph node enlargement with inflammatory signals  Fever | Self-resolving |
| 005 | AFB sputum smear +, sputum culture +, Xpert MTB/RIF^®^ + | Yes | Disseminated | 18 | Left cervical lymph node enlargement with inflammatory signals | Prednisone |
| 040 | ‒ | Yes | Lymph Node | 19 | Right cervical lymph node enlargement with inflammatory signals  Fever | Self-resolving |
| 042 | AFB sputum smear -, Xpert MTB/RIF^®^ + | Yes | Extrapulmonary | 7 | Left posterior cervical lymph node enlargement with inflammatory signals  Fever | Self-resolving |

AFB = acid-fast bacilli;

IRIS = Immune Reconstitution Inflammatory Syndrome.
