# Supplementary material for: Changes in the NK Cell Repertoire Related to Initiation of TB Treatment and Onset of Immune Reconstitution Inflammatory Syndrome in TB/HIV Co-infected Patients in Rio de Janeiro, Brazil—ANRS 12274
Source: Front Immunol. 2019 Aug 13;10:1800. doi: 10.3389/fimmu.2019.01800 (PMC6700218; doi:10.3389/fimmu.2019.01800)
Supplement: Supplementary Table 2 — Medians and interquartile ranges (IQRs) of the circulating NK cell repertoire frequencies. [file Table_2.DOCX]

**Supplementary Table 2. Medians and interquartile ranges (IQRs) of the circulating NK cell repertoire frequencies.**

|  |  |  |  |  |  |
| --- | --- | --- | --- | --- | --- |
| **%NK receptors**  **Median (IQR)** | **TB/HIV**  **(N=33)** | **HIV**  **(N=25)** | **TB**  **(N=27)** | **HC**  **(N=25)** | **P value*** |
| CD94 | 30.25  (16.90-45.42) | 44.09  (27.86-58.30) | 39.23  (24.05-51.40) | 37.46  (25.85-55.71) | 0.027^a^ |
| NKG2A | 14.17  (7.24-25.58) | 18.18  (11.22-28.39) | 22.83  (6.18-35.93) | 23.48  (18.68-36.33) | 0.024^c^ |
| NKG2C | 13.82  (7.56-23.39) | 22.69  (6.01-39.71) | 30.25  (16.9-45.42) | 41.04  (21.31-54.42) | *<*0.0001^c^ |
| NKG2D | 31.13  (17.76-53.23) | 41.77  (21.64-74.89) | 52.82  (24.72-75.43) | 60.08  (34.23-73.14) | 0.0042^c^ |
| CD158a | 13.08  (7.28-24.73) | 22.78  (12.13-47.50) | 13.56  (5.54-29.83) | 14.19  (7.21-23.51) | 0.009^a^ |
| CD158b1/b2,j | 12.16  (6.34-24.07) | 18.73  (7.80-42.74) | 15.74  (12.33-23.89) | 16.56  (10.33-24.18) |  |
| CD158d | 5.55  (3.24-8.43) | 7.43  (2.16-23.94) | 1.66  (0.74-11.16) | 5.27  (2.67-17.36) | 0.019^b^ |
| CD158e | 6.76  (4.52-14.14) | 10.46  (4.60-12.94) | 4.74  (1.54-12.94) | 10.18  (3.80-16.82) |  |
| CD158e1/e2 | 3.60  (1.16-10.72) | 10.33  (4.26-13.55) | 2.13  (1.38-8.20) | 7.29  (2.96-11.7) | 0.026^a^ |
| CD158i | 8.60  (4.79-20.79) | 12.38  (5.25-18.37) | 4.86  (1.84-15.38) | 10.96  (3.40-24.51) |  |
| NKp30 | 9.07  (4.44-18.68) | 15.61  (10.21-25.70) | 18.07  (7.82-45.98) | 23.27  (18.19-28.56) | 0.026^a^  0.019^b^  0.002^c^ |
| NKp44 | 0.76  (0.27-1.77) | 1.08  (0.26-4.05) | 0.53  (0.19-1.77) | 0.73  (0.31-1.28) |  |
| NKp46 | 28.32  (16.48-46.97) | 26.65  (18.18-41.70) | 39.28  (17.52-57.42) | 53.53  (35.78-60.36) | 0.0001^c^ |
| NKp80 | 21.66  (10.84-38.73) | 26.87  (11.06-57.55) | 32.76  (17.00-60.33) | 36.29  (24.28-56.12) |  |
| CD85j | 23.44  (8.84-43.62) | 30.43  (13.14-38.90) | 31.40  (15.18-51.60) | 38.28  (23.97-52.59) | 0.042^c^ |
| CD160 | 12.45  (7.40-17.79) | 16.60  (6.63-21.94) | 10.92  (4.69-16.43) | 6.45  (3.61-22.44) |  |
| CD161 | 25.30  (12.43-8.43) | 25.53  (15.85-47.66) | 39.99  (17.05-64.72) | 52.43  (37.49-60.27) | 0.028^b^  <0.0001^c^ |
| DNAM-1 | 78.61  (66.94-87.79) | 87.38  (73.88-89.79) | 79.02  (59.98-86.99) | 82.48  (75.72-87.39) |  |
| CD244 | 45.63  (20.92-67.34) | 56.41  (44.88-78.24) | 63.98  (41.85-78.88) | 63.67  (45.45-78.62) | 0.048^a^  0.016^b^  0.004^c^ |
| CD69 | 9.07  (4.81-20.61) | 11.16  (3.82-18.39) | 7.48  (2.13-15.86) | 1.31  (0.70-3.93) | <0.0001^c^ |

N = number of cases;

IQR = Interquartile ranges (75th-25th percentiles);

Mann Whitney *U*-test to TB/HIV group: *vs* HIV^a^, *vs* TB^b^, *vs* HC^c^.
